# Associations between Dental Checkups and Unmet Dental Care Needs: An Examination of Cross-Sectional Data from the Seventh Korea National Health and Nutrition Examination Survey (2016–2018)

**DOI:** 10.3390/ijerph18073750

**Published:** 2021-04-03

**Authors:** Jong-Hwa Jang, Ji-Liang Kim, Jae-Hyun Kim

**Affiliations:** 1 Department of Dental Hygiene, College of Health Science, Dankook University, Cheonan-si 31116, Korea; jhj@dankook.ac.kr; 2 Department of Public Health, General Graduate School of Dankook University, Cheonan-si 31116, Korea; 201002698@hanmail.net; 3 Department of Health Administration, College of Health Science, Dankook University, Cheonan-si 31116, Korea

**Keywords:** oral health, dental checkup, Korea National Health and Nutrition Examination Survey (KNHANES), dental care, dental service utilization

## Abstract

To identify gender- and age-related associations between adult dental checkups and unmet dental care needs, we analyzed data of 14,000 participants, from the Seventh Korea National Health and Nutrition Examination Survey (2016–2018). Data were collected via self-report questionnaires and interviews. The complex sample chi-square test and multiple logistic regression analysis indicated that 31.7% of participants had unmet dental care needs. Within the previous 12 months, 56.5% did not undertake dental checkups, and 29.3% did not use a dental service. Odds ratios (ORs) of the unmet dental needs were 8.87 (confidence interval (CI) = 7.80–10.09, *p* < 0.001) for those who did not use dental services and 1.28 (CI = 1.13–1.44, *p* < 0.001) for those who did not have dental checkups. Significant age-dependent associations between those not receiving dental checkups and the rate of unmet dental care included men and women aged 50–59 years and women ≥70 years. However, unmet dental care needs for men aged ≥70 years not undergoing dental checkups were not statistically significant (*p* = 0.311). Overall, it was found that the use of dental service and dental checkups were the influencing factors for unmet dental care needs.

## 1. Introduction

In South Korea, all citizens are obliged to join the National Health Insurance scheme under the National Health Insurance Act (except those with an approved exemption for a specific reason), and the insured and their dependents are entitled to free preventive dental checkups [1]. However, the reported rate of compliance with routine dental checkups is very low (31.0% in 2015; 31.8% in 2017) compared to general health examinations (76.1% in 2015; 78.5% in 2017) [2,3].

Regular dental checkups make early detection of any dental health issues possible, thus preventing them from developing into critical problems and minimizing the cost and time necessary for treatment [4,5]. Adults with risk factors (e.g., smoking and diabetes) can reduce the incidence of tooth loss by receiving dental checkups every 6 months [6]. Furthermore, regular dental checkups provide dental service users with an accurate evaluation of their oral health status, enhancing their motivation to prevent and treat oral diseases [7,8]. Cho et al. [9] reported that mothers who received dental checkups are twice as likely to have their children treated with pit and fissure sealant compared to mothers who did not. The main factor contributing to unmet dental care needs is that those who do not receive regular dental checkups cannot know their accurate dental health status and often fail to recognize their dental problems unless accompanied by pain [10]. In particular, a study on the unmet dental care needs among older adults (≥65 years) reported that 75% of them had no experience of dental checkup [1].

Having unmet dental care needs is a state of not being able to acquire timely dental treatment [10]. According to the Organization for Economic Cooperation and Development (OECD), the wide mean rate of unmet dental care needs has been gradually decreasing, falling from 10% in 2015 and 8.7% in 2016 to 6.0% in 2017 [11]. Against this global trend, the rate of unmet dental care needs in South Korea has remained at a very high level: 29.7% in 2013, 32.4% in 2014, and 32.2% in 2015. That is, almost one-third of the Korean population does not receive dental care despite their perceived needs [9]. The most frequent reason for this, as found in the Korea National Health and Nutrition Examination Survey (KNHANES) VI, is “economic burden”, followed by being considered as “less important than other problems” [12]. However, above all, in order to resolve unmet dental care needs, it will be important for patients to accurately recognize their oral health status and receive dental treatment. Designing an adult oral health promotion program must be preceded by determining the current status of dental checkups and unmet dental care needs. The KNHANES is a nationwide cross-sectional survey providing representative and reliable data on the health and nutritional status of the general population. The purpose of this study was to identify the current state and related factors regarding the unmet dental care needs perceived during the past 12 months by Korean adults aged 19 years or older, using KNHANES VII (2016–2018) datasets, and to identify the association between dental checkups and unmet dental care needs for different genders and age groups.

## 2. Materials and Methods

### 2.1. Study Design and Participants

This study analyzed the association between dental checkups and unmet dental care needs among Korean adults using the KNHANES VII. Data files for KNHANES datasets are available for public use [13]. The survey participants were selected by complex sampling with progressively applied proportional allocation and systematic sampling. Interviewers were trained with respect to KNHANES survey guidelines. They conducted interviews using structured questionnaire items after obtaining consent from participants.

The data for KNHANES VII used in this study were collected from the selected survey population based on the 2010 Population and Housing Census; the basic sampling framework was supplemented with the declared values of public housing to include the latest information that reflects the characteristics of the current population. Sampling was performed using a method based on a two-stage stratified clustering method, whereby the enumerations of the districts and the households are considered as the primary and secondary sampling units, respectively. KNHANES VII stratified the sampling framework according to the divisions of si-do (municipality/province, the largest administrative units in Korea), dong-eup-myeon (enumeration of districts, the smallest administrative units in municipality and province), and housing type (detached and multifamily). Variables such as the ratio of residential area and the education level of the householder were used as the criteria for implicit stratification. From the first year (2016), 192 enumerated districts were selected as the primary sampling units; 23 appropriate households were sampled from each of the districts using a systematic sampling method that excluded facilities such as nursing homes, military barracks, prisons, and foreign households. In each sample household, all household members over the age of 1 year, considered to meet the requirements of appropriate household members, were selected as survey participants. 

**Figure 1:**
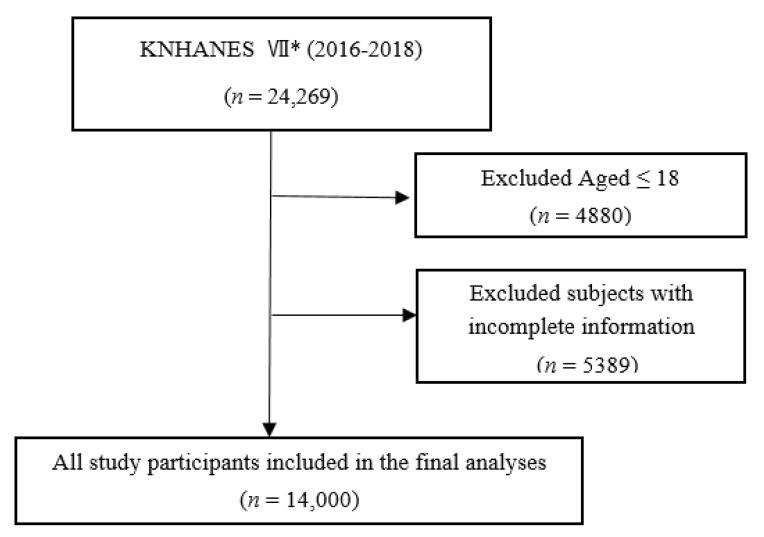
Flow chart of the study population. * The Seventh Korea National Health and Nutrition Examination Survey.

After excluding those aged 18 years or younger (*n* = 4880) and those who provided incomplete information (*n* = 5389) from the study population of 24,269 (8150 in 2016, 8127 in 2017, and 7992 in 2018), 14,000 participants were selected for final analysis (Figure 1).

### 2.2. Variables and Statistical Analysis

A complex sample chi-square test was performed to reflect the respondents′ characteristics to unmet dental care needs and the association between general characteristics of the participants on unmet dental care needs. Complex sample multiple logistic regression analysis was performed to determine the association between dental checkups and unmet dental care needs. Additionally, complex sample multiple logistic regression analysis was performed to determine the association between dental checkups and unmet dental care needs, which was also analyzed by gender and age. Statistical analysis was performed using SAS software 9.4 (SAS Institute Inc., Cary, NC, USA). 

The survey consisted of questions on the participants’ sociodemographic characteristics, unmet dental care needs, participation in dental checkups, utilization of dental services, perceived health status, and health-related behavior. The variable selection was included on the basis of various previous studies on the factors affecting dental checkups and unmet dental care needs [1,4,5,10,12,14].

The independent variable was dental checkup, specifically whether the participant underwent dental checkups within the previous 12 months, which was quantified by assigning a score to the response (yes/no) to the following question: “Within the previous 12 months, have you ever received a dental checkup to find out your oral health status?” [5]. The utilization of dental clinic service was quantified by assigning a score to the response (yes/no) to the following question: “Within the previous 12 months, have you ever visited any dental clinic?” [15]. 

The dependent variable was defined as the unmet dental care needs, which was quantified by assigning a score to the response (yes/no) to the following question: “Within the previous 12 months, have you perceived a need for dental care (examination or treatment) but could not receive it?” [10]. 

Sociodemographic variables were set as confounding variables: region (Seoul, Metropolitan City, and other), gender (male and female), age (19–29, 30–39, 40–49, 50–59, 60–69, and ≥70 years), household income (low, lower–middle, upper–middle, and high), education level (elementary school, middle school, high school, and post-secondary education), and marital status (married and single). Variables related to health behavior included alcohol consumption (no/yes), smoking status (current smoker, past smoker, and nonsmoker), and walking days per week (never, 1–2, 3–4, 5–6, and every day). Perceived health (good, moderate, bad) was defined as the variable related to health status.

Although KNHANES VII, as a survey conducted by the government for public benefits pursuant to the Bioethics and Safety Act, was exempted from review by the Institutional Review Board (IRB) of the Centers for Disease Control and Prevention, this study was approved by the IRB of Dankook University (DKU: 2020-08-009) for ethical considerations. In addition, the authors followed the guidelines of the Declaration of Helsinki.

## 3. Results

### 3.1. Effects of General Characteristics on the Unmet Dental Care Needs within the Previous 12 Months

Table 1 outlines the analysis results of the unmet dental care needs perceived within the previous 12 months, according to the participants′ general characteristics. Of the study population, 31.7% had unmet dental care needs, while 56.5% did not receive dental checkups, of whom 43.3% perceived unmet dental care needs (*p* < 0.001). Within the previous 12 months, 29.3% did not use a dental service, of whom 67.1% had unmet dental care needs (*p* < 0.001).

The effects of general characteristics of the participants on their unmet dental care needs were as follows: women had a higher rate of unmet dental care needs than men (34.0% vs. 29.2%) (*p* < 0.001); the older the participant, the higher the unmet dental care needs (≥70 years: 34.4%) (*p* < 0.001); the lower the income and education levels, the higher the unmet dental care needs (*p* < 0.001); married participants had a higher rate of unmet dental care needs than single participants (32.6% vs. 28.2%) (*p* < 0.001); perceived health status was associated with unmet dental needs in increasing order of “good” (24.0%), “moderate” (31.5%), and “bad” (44.1%) (*p* < 0.001); smoking status was associated with unmet dental care needs in increasing order of past smokers (27.4%), nonsmokers (31.2%), and current smokers (37.4%) (*p* < 0.001); the frequency of walking days per week was inversely related with unmet dental care needs in increasing order (never: 39.5% to every day: 27.9%) (*p* < 0.001).

### 3.2. Association between Dental Checkups and Unmet Dental Care Needs

Table 2 outlines the results of the logistic regression analysis of the association between dental checkups and unmet dental care needs according to sociodemographic characteristics, perceived health status, and health behavior. 

Model 1 was adjusted for all variables excluding “dental service utilization within the previous 12 months”; the odds ratio (OR) of those who did not receive dental checkups had a higher rate of unmet dental care needs (OR = 3.64; confidence interval (CI): 3.30–4.02; *p* < 0.001). Model 2 was adjusted for all variables excluding “dental checkups within the previous 12 months”; the OR of those who did not utilize dental service concerning the rate of unmet dental care needs was 10.04 (CI = 8.95–11.25; *p* < 0.001). Model 3 was adjusted for all variables; the OR of those who did not utilize dental service for the rate of unmet dental care needs was 8.87 (CI = 7.80–10.09; *p* < 0.001); the OR of those who did not receive dental checkups with respect to the rate of unmet dental care was 1.28 (CI = 1.13–1.44; *p* < 0.001).

### 3.3. Association between Dental Checkups and Unmet Dental Care Needs by Gender and Age

Table 3 outlines the results of the logistic regression analysis of the association between dental checkups and unmet dental care needs by gender and age.

In Model 1, the OR ranged between 1.74 and 4.89 (*p* < 0.001) for men and between 2.94 and 5.49 for women, (*p* < 0.001). In Model 2, the unmet dental needs were significantly higher in men in their 50s (OR = 1.94, CI = 1.33–2.82, *p* = 0.001), and the ORs of the three female age groups (40s, 50s, and 70s) were statistically significant, ranging from 1.57 to 1.99 (*p* < 0.05). In Model 3, men in their 50s had a higher OR (1.77; CI = 1.22–2.58, *p* = 0.003), and significant differences were observed in women aged 70s years or older (OR = 1.79, CI = 1.20–2.66, *p* = 0.004) and in their 50s (OR: 1.43; CI: 1.02–2.02; *p* = 0.040). What is noteworthy with regard to gender differences is that a significant association between dental checkups and unmet dental care needs was observed in women aged 70 years or older, but not in their male counterparts (*p* = 0.311).

## 4. Discussion

This study analyzed the association between dental checkups and unmet dental care needs using the KNHANES VII datasets. The rate of unmet dental care needs among Korean adults was calculated as 31.7%, a moderate rise from the rate drawn from the KNHANES VI datasets (27.4%) [12]. An increase in unmet dental care needs means an increase in the prevalence of oral diseases due to the failure to provide timely treatment, exacerbated over time. This problem is particularly detrimental in Korea, where single-member households are increasing, including the number of vulnerable populations such as older adults and people with disabilities [3]. Older adults living alone without caregivers or individuals with physical and economic vulnerabilities are at a higher risk of having unmet dental care needs [16,17]. 

Many studies have been conducted on the causes of unmet dental care needs among Koreans: sociodemographic characteristics [18], factors related to dental pain [14], insecurity in the employment market [19,20], the Decayed, Missing, and Filled Teeth (DMFT) index [21], and geographic accessibility [17]. In general, economic burden was found to be the most frequent cause of unmet dental care needs [16,22]. Jun and Ryu [23] reported that Koreans do not visit dental clinics primarily for economic reasons; Moon and Song [12] reported similar findings. Although Korea has a well-developed health insurance system, dental care often involves dental prostheses (e.g., crowns and bridges), which are not covered by the insurance, and the coverage for implants and dentures is limited to 65 years of age or older, thus imposing a considerable burden on the budget of many people in need of dental care [17,18]. Since early-phase dental treatment and preventive care are mostly covered by insurance with affordable copays and oral diseases are not cured spontaneously—and worsen when left unattended—preventive care (before disease occurs) is important [24]. The first step in preventive care involves regular dental checkups [25]. A dental checkup not only provides an accurate diagnosis of untreated teeth but also promotes and maintains oral health by inducing proper dental treatment.

Previous studies on unmet medical needs in Korea [26,27] reported that a higher rate of unmet medical needs was associated with the absence of a spouse, low household income or education level of the householder, medical aid recipients, chronic diseases, and poor perceived health status. In this study, no significant association was found between unmet dental care needs and presence/absence of the spouse. However, the association with household income, education level, and perceived dental health status was also verified in this study. Regarding the association with health behavior, the unmet dental care needs of current smokers was found to be 1.49-fold higher compared to nonsmokers, and alcohol consumption was found to be inversely associated with unmet dental care needs.

Poor oral health, such as chronic periodontal disease and an increasing number of missing teeth, can lead to nutritional imbalance due to impaired masticatory function and discomfort associated with food intake, which may affect systemic health [23]. 

The analysis of the factors that affect unmet dental care needs after adjusting for the participants’ general characteristics revealed that, within the previous 12 months, the OR of those who did not utilize dental service was 8.87, and that of those who did not receive dental checkups was 1.28. Jeon et al. [15] pointed out that one of the characteristics of people with unmet dental care needs is that they did not receive dental checkups within the previous 12 months. Regular dental checkups help people have a clear overview of their oral health conditions, along with increased knowledge of periodontal health [28] and awareness of oral health problems, thus increasing the rate of patients who undergo yearly visits for dental care [3,15]. In contrast, unmet dental care needs may occur for people who do not receive regular dental checkups due to failure in recognizing their oral health conditions. 

In this study, the rate of dental checkups was 43.5%, a significant increase compared to 30.1% in the second year of KNHANES VI (2014) [25]. This may be ascribed to an increase in people’s knowledge of oral health with easy access to related information due to the rapid development of social culture digitization. However, according to a study by Hwang et al. [4], 76.3% of Korean adults did not visit dental clinics for checkup purposes or had no intention of receiving dental checkups within the next 6 months. In addition, compared with the general health checkup rate of 78.5% (as of 2017) [2], the dental checkup rate is still significantly lower. As one of the causes of the low dental checkup rate, a low recognition of the need for regular dental checkups can be assumed [5]. Schouten et al. [29] noted that preferences for regular dental checkups basically arise from one’s intrinsic motivation for maintaining oral health. Choi [8] pointed out that the high dental checkup experience rate (83.5%) among the working population was attributable to the fact that checkups were performed at the workplace, highlighting the importance of geographical and economic accessibility to dental checkups. From the results of studies on the association between the rate of unmet dental care needs and prevalence factors [30,31,32,33], it can be assumed that it is necessary to provide citizens with facilities for dental checkups in their neighborhood and gradually expand them to increase the rate of dental checkups and lower the rate of unmet dental care needs. For older adults and people with disabilities with limited mobility, it is also necessary to establish a visiting dental care service scheme in which dentists or dental hygienists visit and perform dental checkups. Furthermore, oral health education programs should be reinforced to enhance the motivation for compliance with regular dental checkups by actively informing people of their importance.

The results of this study confirm the association between various factors affecting unmet dental care needs, thus verifying the importance of dental service utilization and regular dental checkups. In particular, the rate of unmet dental care needs in men and women in their 50s was 1.77 and 1.43 times higher, respectively, when dental checkups were not performed. This highlights the urgency of preparing strategies for enhancing the rate of dental checkups. In the older adults (≥70 years), however, the rate was 1.79 times higher in women, but no significant differences were observed in men, which implies the need to set up and implement oral health promotion strategies taking into account gender and age characteristics. A strong point of this study is the generalizability of its results because it employed a complex sample design using pertinent datasets from the large-scale KNHANES conducted nationwide. However, as a limitation of this study, it may be pointed out that, due to the nature of KNHANES VII as a cross-sectional survey, a temporal relationship could not be traced in the association between dental checkups and the rate of unmet dental care needs. Future research will have to investigate the effect of dental service utilization and dental checkups on oral health behavior and oral health status, as well as trace the trend of the factors affecting unmet dental care need by conducting longitudinal research.

## 5. Conclusions

This study revealed that three out of 10 (31.7%) Korean adults had unmet dental care needs. In particular, within the previous 12 months, 43.3% of those who did not receive dental checkups and 67.1% of those who did not use dental service had unmet dental care needs. The factors affecting unmet dental care needs were nondental service utilization (OR = 8.87) and nondental checkups (OR = 1.28). In addition, unmet dental care needs were most associated with both men and women in their 50s and with women in their 70s who did not have dental checkups (*p* = 0.004). Therefore, it is necessary to prepare an effective strategy to increase dental service utilization in order to reduce unmet dental care needs for everyone. In this regard, further studies are required to identify various determinants related to unmet dental care needs according to sex and age.

## Figures and Tables

**Table 1:** Unmet dental care needs according to general characteristics of subjects.

Variables	Division	Total	Yes	No	*p*-Value **
		*n* (% *)	*n* (% *)	*n* (% *)	
Unmet dental care needs	All	14,000 (100.0)	4561 (31.7)	9439 (68.3)	
Dental checkups	No	8056 (56.5)	3555 (43.3)	4501 (56.7)	<0.001
	Yes	5944 (43.5)	1006 (16.6)	4938 (83.4)	
Dental service utilization	No	4062 (29.3)	2801 (67.1)	1261 (32.9)	<0.001
	Yes	9.938 (70.7)	1760 (17.0)	8178 (83.0)	
Area	Seoul	2690 (19.4)	814 (29.9)	1876 (70.1)	0.058
	Metropolitan city	3666 (27.8)	1157 (30.6)	2509 (69.4)	
	Other	7644 (52.8)	2590 (32.9)	5054 (67.1)	
Sex	Male	5998 (48.6)	1761 (29.2)	4237 (70.8)	<0.001
	Female	8002 (51.4)	2800 (34.0)	5202 (66.0)	
Age	19–29	1559 (16.8)	415 (25.1)	1144 (74.9)	<0.001
	30–39	2089 (17.1)	705 (33.8)	1384 (66.2)	
	40–49	2625 (20.8)	864 (32.8)	1761 (67.2)	
	50–59	2811 (21.2)	910 (31.7)	1901 (68.3)	
	60–69	2524 (13.4)	853 (33.1)	1671 (66.9)	
	≥70	2392 (10.7)	814 (34.4)	1578 (65.6)	
Household income	Low	2697 (15.8)	1058 (38.5)	1639 (61.5)	<0.001
	Middle–low	3345 (23.2)	1264 (37.9)	2081 (62.1)	
	Middle–high	3791 (29.0)	1224 (32.2)	2567 (67.8)	
	High	4167 (32.0)	1015 (23.3)	3152 (76.5)	
Education	Elementary	2856 (14.4)	1058 (38.5)	1711 (59.5)	<0.001
	Middle	1442 (8.9)	1264 (37.9)	900 (63.7)	
	High	4472 (35.0)	1224 (32.2)	3.030 (68.1)	
	University or high	5230 (41.7)	1015 (23.3)	3.798 (72.6)	
Marriage	Married	11,804 (78.0)	3909 (32.6)	7895 (67.4)	<0.001
	Single	2196 (22.0)	652 (28.2)	1544 (71.8)	
Perceived health	Good	3850 (29.1)	946 (24.0)	2904 (76.0)	<0.001
	Moderate	7260 (52.3)	2334 (31.5)	4926 (68.5)	
	Bad	2890 (18.6)	1281 (44.1)	1609 (55.9)	
Drinking	No	1579 (9.0)	538 (33.1)	1041 (66.9)	0.282
	Yes	12,421 (91.0)	4023 (31.5)	8398 (68.5)	
Smoking	Current smoker	2492 (21.3)	958 (37.4)	1534 (62.6)	<0.001
	Past smoker	3023 (21.6)	841 (27.4)	2182 (72.6)	
	Nonsmoker	8485 (57.1)	2762 (31.2)	5723 (68.8)	
Walking days per week	Never	2707 (17.7)	1073 (39.5)	1634 (60.5)	<0.001
	1–2	2331 (16.7)	786 (33.5)	1545 (66.5)	
	3–4	2747 (19.7)	842 (30.0)	1905 (70.0)	
	5–6	2337 (17.6)	724 (30.0)	1613 (70.0)	
	Everyday	3878 (28.3)	1.136 (27.9)	2742 (72.1)	

* Weighted %; ** *p*-value was calculated using a complex sample chi-square test.

**Table 2:** Factors affecting unmet dental care needs.

Variables	Model 1		Model 2		Model 3	
	OR (95% CI)	*p*-Value *	OR (95% CI)	*p*-Value *	OR (95% CI)	*p*-Value *
Dental checkups						
No	3.64 (3.30–4.02)	<0.001			1.28 (1.13–1.44)	<0.001
Yes	1.00				1.00	
Dental service utilization						
No			10.04 (8.95–11.25)	<0.001	8.87 (7.80–10.09)	<0.001
Yes			1.00		1.00	
Area						
Seoul	1.04 (0.93–1.18)	0.494	1.22 (1.07–1.38)	0.003	1.22 (1.07–1.38)	0.003
Metropolitan city	0.95 (0.85–1.07)	0.413	0.87 (0.75–1.06)	0.061	0.88 (0.76–1.01)	0.075
Other	1.00		1.00		1.00	
Sex						
Male	0.73 (0.64–0.82)	<0.001	0.68 (0.60–0.78)	<0.001	0.68 (0.60–0.78)	<0.001
Female	1.00		1.00		1.00	
Age						
19–29	1.07 (0.82–1.39)	0.644	0.97 (0.72–1.31)	0.836	0.97 (0.72–1.31)	0.856
30–39	1.68 (1.37–2.06)	<0.001	1.28 (1.01–1.61)	0.042	1.30 (1.03–1.64)	0.030
40–49	1.71 (1.41–2.07)	<0.001	1.30 (1.05–1.61)	0.016	1.34 (1.08–1.66)	0.008
50–59	1.46 (1.22–1.74)	<0.001	1.34 (1.10–1.64)	0.003	1.36 (1.12–1.66)	0.002
60–69	1.31 (1.12–1.53)	0.001	1.25 (1.05–1.48)	0.012	1.27 (1.07–1.51)	0.006
≥ 70	1.00		1.00		1.00	
Household income						
Low	1.50 (1.28–1.75)	<0.001	1.46 (1.22–1.74)	<0.001	1.43 (1.19–1.71)	<0.001
Middle–low	1.64 (1.45–1.87)	<0.001	1.51 (1.31–1.73)	<0.001	1.50 (1.30–1.72)	<0.001
Middle–high	1.39 (1.23–1.56)	<0.001	1.36 (1.19–1.54)	<0.001	1.39 (1.18–1.54)	<0.001
High	1.00		1.00		1.00	
Education						
Elementary	1.11 (0.94–1.33)	0.226	1.30 (1.07–1.58)	0.010	1.25 (1.02–1.52)	0.031
Middle	1.06 (0.87–1.28)	0.568	1.16 (0.93–1.45)	0.176	1.13 (0.91–1.41)	0.264
High	1.05 (0.94–1.17)	0.390	1.07 (0.96–1.21)	0.233	1.06 (0.95–1.20)	0.307
University	1.00		1.00		1.00	
Marriage						
Married	1.02 (0.86–1.21)	0.857	1.04 (0.85–1.27)	0.718	1.04 (0.85–1.27)	0.688
Single	1.00		1.00		1.00	
Perceived health						
Good	0.49 (0.43–0.56)	<0.001	0.45 (0.39–0.53)	<0.001	0.46 (0.39–0.53)	<0.001
Moderate	0.63 (0.56–0.70)	<0.001	0.57 (0.50–0.65)	<0.001	0.57 (0.50–0.65)	<0.001
Bad	1.00		1.00		1.00	
Alcohol consumption						
No	0.89 (0.76–1.04)	0.129	0.81 (0.68–0.97)	0.020	0.81 (0.68–0.97)	0.019
Yes	1.00		1.00		1.00	
Smoking						
Smoker	1.39 (1.20–1.61)	<0.001	1.50 (1.07–1.38)	<0.001	1.49 (1.28–1.77)	<0.001
Past smoker	0.99 (0.86–1.15)	0.935	1.11 (0.95–1.30)	0.204	1.11 (0.95–1.30)	0.235
Nonsmoker	1.00		1.00		1.00	
Walking days per week						
Never	1.28 (1.11–1.47)	0.001	1.20 (1.07–1.38)	0.027	1.19 (1.02–1.40)	0.036
1–2	1.15 (1.00–1.32)	0.043	1.15 (1.07–1.38)	0.070	1.15 (0.99–1.34)	0.081
3–4	1.03 (0.89–1.19)	0.735	1.02 (1.07–1.38)	0.834	1.01 (0.87–1.20)	0.879
5–6	1.10 (0.96–1.27)	0.163	1.08 (1.07–1.38)	0.339	1.08 (0.92–1.26)	0.319
Everyday	1.00		1.00		1.00	

OR = adjusted odds ratios, CI = confidence interval; * *p*-value was calculated using a complex sample multiple logistic regression; Model 1 was adjusted for dental checkups, area, gender, age, household income, education, marriage, residence, perceived health, alcohol consumption, smoking, and walking days per week excluding dental service utilization; model 2 was adjusted for dental service utilization, area, gender, age, household income, education, marriage, residence, perceived health, alcohol consumption, smoking, and walking days excluding dental checkups; model 3 was adjusted for all variables; dependent variable: unmet dental care needs.

**Table 3:** Association between unmet dental care needs and use of dental checkups according to sex and age.

Variables		Model 1		Model 2		Model 3	
Sex	Age, Years	OR (95% CI)	*p*-Value *	OR (95% CI)	*p*-Value *	OR (95% CI)	*p*-Value *
Male	All	3.39 (2.91–3.95)	<0.001	1.32 (1.09–1.59)	0.005	1.22 (1.01–1.48)	0.038
	19–29	3.13 (1.97–4.96)	<0.001	1.06 (0.60–1.90)	0.834	0.85 (0.47–1.52)	0.572
	30–39	4.89 (3.37–7.10)	<0.001	1.56 (0.98–2.49)	0.061	1.51 (0.92–2.47)	0.104
	40–49	3.28 (2.44–4.40)	<0.001	1.06 (0.72–1.56)	0.752	1.00 (0.67–1.49)	0.982
	50–59	4.25 (3.11–5.81)	<0.001	1.94 (1.33–2.82)	0.001	1.77 (1.22–2.58)	0.003
	60–69	3.30 (2.33–4.66)	<0.001	1.51 (1.00–2.27)	0.050	1.32 (0.88–1.98)	0.182
	≥70	1.74 (1.15–2.65)	0.009	0.84 (0.52–1.34)	0.461	0.77 (0.47–1.27)	0.311
Female	All	4.29 (3.80–4.85)	<0.001	1.49 (1.28–1.72)	0.001	1.30 (1.12–1.52)	0.001
	19–29	3.65 (2.49–5.34)	<0.001	1.11 (0.68–1.82)	0.670	1.04 (0.63–1.71)	0.874
	30–39	5.23 (3.85–7.11)	<0.001	1.03 (0.67–1.59)	0.883	0.94 (0.62–1.44)	0.788
	40–49	5.49 (4.19–7.18)	<0.001	1.57 (1.10–2.23)	0.014	1.40 (0.98–2.02)	0.068
	50–59	4.58 (3.59–5.85)	<0.001	1.68 (1.23–2.30)	0.001	1.43 (1.02–2.02)	0.040
	60–69	2.94 (2.20–3.94)	<0.001	1.24 (0.90–1.71)	0.180	1.13 (0.81–1.56)	0.473
	≥70	3.92 (2.77–5.54)	<0.001	1.99 (1.36–2.92)	<0.001	1.79 (1.20–2.66)	0.004

OR = adjusted odds ratios, CI = confidence interval; * *p*-value was calculated using a complex sample multiple logistic regression; Model 1 was unadjusted; model 2 was adjusted for dental service utilization; model 3 was adjusted for dental service utilization, area, household income, education, marriage, residence, perceived health, alcohol consumption, smoking, and walking days per week; dependent variable: unmet dental care needs.

## Data Availability

The data presented in this study are available on reasonable request from the corresponding author.

## References

[1] Park S.Y. (2018). Factors affecting the rate of oral examination in the elderly in local communities. J. Korean Soc. Dent. Hyg..

[2] Social Security Committee General Health Examination Rate. http://ssc.go.kr/stats/infoStats/stats010100_view.do?indicator_id=486&listFile=stats010200&chartId=2005.

[3] KOSIS (Korean Statistical Information Service) Health Checkup Statistics: Status of the Subjects and the Number of Examinees by Gender by Trial. http://kosis.kr/statHtml/statHtml.do?orgId=350&tblId=DT_35007_N035&vw_cd=MT_ZTITLE&list_id=350_35007_A005&seqNo=&lang_mode=ko&language=kor&obj_var_id=&itm_id=&conn_path=MT_ZTITLE.

[4] Hwang S.H., Choi H.S., Son S.H. (2011). Original: A Survey on Oral Examination Behavior of Adults in Their 20s Based on Transtheoretical Model. J. Dent. Hyg. Sci..

[5] Kim D.H., Seo Y.J. (2017). Factors influencing the regular oral check-ups: Based on the data of the 2014 Korea national health and nutrition examination survey. J. Dent. Hyg. Sci..

[6] Hahn T.W., Kraus C., Hooper-Lane C. (2017). Clinical inquiries: What is the optimal frequency for dental checkups for children and adults?. J. Fam. Pract..

[7] Shin H.S., Ahn E.S. (2017). Effectiveness of oral examination for infants and toddlers: Effects on subsequent utilization and costs. J. Korean Acad. Oral Health.

[8] Choi M.H. (2010). A survey research on industrial workers’ oral examination status and oral health educational request level. J. Korean Acad. Dent. Hyg..

[9] Cho Y.S., Chun K.H., Baek K.W., Kim M.S., Lee S.J. (2012). The relationship of pit and fissure sealant in children and mother’s socioeconomic status, mother’s oral-health screening. J. Korean Acad. Oral Health.

[10] Yoo S.H., Park I.S., Kim Y.M. (2017). A decision-tree analysis of influential factors and reasons for unmet dental care in Korean adults. Health Soc. Welf. Rev..

[11] European Health Statistics Unmet Health Care Needs Statistics. https://ec.europa.eu/eurostat/web/products-datasets/-/hlth_silc_22.

[12] Moon S.E., Song A.H. (2016). Factors affecting unmet dental care needs of Korean: The 6th Korean national health and nutritional examination survey. J. Korean Soc. Dent. Hyg..

[13] Korea Centers for Disease Control and Prevention. Korea National Health and Nutrition Examination Survey (KNHANES VII). http://knhanes.cdc.go.kr.

[14] Ahn E.S., Shin M.S. (2016). Factors related to the unmet dental care needs of adults with dental pain. J. Dent. Hyg. Sci..

[15] Jeon J.E., Chung W.G., Kim N.H. (2011). Determinants for dental service utilization among Koreans. J. Korean Acad. Oral Health.

[16] Kim J.W., Bae H.J. (2019). A study of the experience of unmet dental care needs among older adults. Health Soc. Welf. Rev..

[17] Yeo J.Y., Jeong H.S. (2012). Determinants of dental screening and unmet dental needs: Interaction effect between geographical accessibility and economic afford ability. Korean J. Health Econ. Policy.

[18] Kim N.H., Chung W.G., Jeon J.E. (2012). The reason of unmet dental need related socioeconomic status in Korea: Using the 4th Korea national health and nutritional examination survey. J. Korean Acad. Oral Health.

[19] Che X.H., Park H.J. (2018). The relationship between precarious work and unmet dental care needs in South Korea: Focus on job and income insecurity. J. Korean Acad. Oral Health.

[20] Kang J.H., Kim C.W., Kim C.S., Seo N.K. (2015). Unmet dental care needs according to employment status. J. Korean Acad. Oral Health.

[21] Chung S.Y., Cho J.W., Jung Y.S., Kim H.Y., Kim J.Y., Choi Y.H., Song K.B. (2017). Association between unmet needs for dental treatment and the DMFT index among Korean adults. J. Korean Acad. Oral Health.

[22] Kim Y.G., Kim E.J., Nho S.H., Baek E.J., Shin M.S., Hwang S.J. (2015). Some adults’ opinions about private dental insurance and national dental insurance according to stress of dental treatment cost. J. Dent. Hyg. Sci..

[23] Jun M.J., Ryu S.Y. (2016). Oral health and behavior by tooth loss: The sixth Korea national health and nutrition examination survey. J. Korea Entertain. Ind. Assoc..

[24] Gładczuk J., Kleszczewska E., Bojko O., Shpakou A., Modzelewska B. (2019). Assessment of socio-health determinants of dental check-ups among students of selected Polish, Belarusian and Ukrainian universities. Oral Health Prev. Dent..

[25] Jin S.B. (2016). Working knowledge of National Health Insurance in dental clinic: Dental records and the receipt book. J. Korean Dent. Assoc..

[26] Kim S.A., Seo Y.W., Woo K.S., Shin Y.J. (2019). A systematic review of studies on current status and influencing factors of unmet medical needs in Korea. J. Crit. Soc. Welf..

[27] Yoon Y.S., Jung B., Kim D., Ha I.H. (2019). Factors underlying unmet medical needs: A cross-sectional study. Int. J. Environ. Res. Public Health.

[28] Varela-Centelles P., Diz-Iglesias P., Estany-Gestal A., Blanco-Hortas A., Bugar B-Gonz Bugar BugSeoane-Romero J.M. (2020). Regular dental attendance and periodontal health knowledge: A cross-sectional survey. Oral Dis..

[29] Schouten B.C., Mettes T.G., Weeda W., Hoogstraten J. (2006). Dental check-up frequency: Preferences of Dutch patients. Community Dent. Health.

[30] Lee M.K., Jin H.J. (2015). The prevalence and association factors of unmet dental care needs in Korean adults: The 5th Korea National Health and Nutritional Examination Survey. J. Korean Soc. Dent. Hyg..

[31] Kim S.Y., Park J., Ryu S.Y., Choi S.W. (2020). Factors of unmet dental care needs among oral health problems and dental care patients. J. Health Inform. Stat..

[32] Choi Y.H., Jin H.J., Kim E.K., Kim B.I., Kim D.K., Park D.Y. (2013). Comparison between the National Oral Health Survey systems of the United States and South Korea. J. Korean Acad. Oral Health.

[33] Ahn E.S., Han J.H. (2015). Measure of unmet dental care needs among Korean adolescent. J. Dent. Hyg. Sci..

